# A scoping review of national policies for dementia prevention and control in mainland China

**DOI:** 10.1186/s12961-025-01314-y

**Published:** 2025-04-03

**Authors:** Jingbin Zhang, Xiaowan Wang, Shasha Yuan

**Affiliations:** Institute of Medical Information/Medical Library, Chinese Academy of Medical Sciences & Peking Union Medical College, Beijing, 100020 China

**Keywords:** China, Dementia, Health policy, Public health, Review

## Abstract

**Introduction:**

Limited knowledge has been found on the policies of dementia prevention and control from low- and middle-income (LMIC) countries. This study aims to provide comprehensive evidence of policy progress regarding dementia prevention and control in mainland China by scoping review on the basis of seven priority areas proposed in The Global Action Plan on the Public Health Response to Dementia 2017–2025.

**Methods:**

We searched the websites of China State Council, National Health Commission, Ministry of Civil Affairs, National Development and Reform Commission, National Healthcare Security Administration and National Administration of Traditional Chinese Medicine for all policies regarding dementia. The keywords included *Chi Dai* (dementia), *A Er Ci Hai Mo* (Alzheimer’s), *Ren Zhi Zhang Ai* (cognitive impairment) and *Shi Zhi* (dementia), and the search covered materials published by 15 April 2023. Policy diffusion analysis, policy reference network analysis and thematic framework analysis were used to analyse the policy contents.

**Results:**

The number of national policies for dementia prevention and control in mainland China increased significantly during 2015–2022. The National Health Commission was responsible for the most policies, while the State Council was responsible for the most important ones. A total of 50 departments were involved in the development of policies for dementia prevention and control, but the stable collaborative relationship among them needs further strengthened. In terms of WHO dementia global action plan, the topic of “dementia as a public health priority” was strongly emphasized, while the areas of “dementia research and innovation” and “information systems for dementia” were less focussed on.

**Conclusions:**

Some policy gaps, including priority arrangement, multisectoral cooperation and policy implementation, must still be addressed in the future to support the interests of people with dementia and their caregivers more effectively.

**Supplementary Information:**

The online version contains supplementary material available at 10.1186/s12961-025-01314-y.

## Introduction

According to the World Social Report 2023 produced by the United Nations, the global population aged 65 years or older is expected to increase from 761 million in 2021 to 1.6 billion in 2050, with the increase in the number of people aged 80 years or older being even more rapid [1]. In 2019, the number of people with dementia (PwD) worldwide was estimated to be 57.4 million, with this figure projected to increase to 83.2 million in 2030 and 152.8 million in 2050 [2]. The global cost of dementia was estimated to be $1.3 trillion in 2019 and is expected to reach $2.8 trillion by 2030 [3]. The complex implications of dementia pose significant challenges to healthcare systems.

Preventing and controlling dementia is a top policy priority worldwide. Various policy documents have been issued by countries and international organizations to address this issue. In 2012, Alzheimer’s Disease International and the WHO jointly published Dementia: A Public Health Priority to encourage the development of policies and the implementation of dementia health plans [4]. The Global Action Plan on the Public Health Response to Dementia 2017–2025 was regarded as a significant step in promoting the wellbeing of PwD and their caregivers [5]. Furthermore, the Decade of Healthy Ageing: Plan of Action [6] emphasized the crucial role of policy change in promoting healthy ageing among PwD. In 2021, the Global Status Report on the Public Health Response to Dementia [3] reported that 34 countries have implemented national dementia plans. However, more services were provided in high-income countries, while scant evidence of such support was found in low- and middle-income (LMIC) countries. The lack of analysis of policy progress on dementia prevention and control further hinders their implementation in practice.

In China, 13 million people were reported to have dementia in 2019, accounting for 25.5% of the global total [7], which is expected to be more than 20 million by 2030 [8]. Providing optimal care to PwD has become a policy priority in the national health agenda. In response to the health challenges posed by chronic diseases and population ageing, and with the goal of enhancing the overall health of the populace, China has issued the Outline of “Healthy China 2030”. This outline aims to promote psychological health and care services for elderly individuals, including effective interventions for people with dementia [9]. Notably, China has designated the prevention and control of dementia as a crucial task within the policy framework. This has provided a clear and forward-looking direction for dementia prevention and control. The Notice on Exploring Special Services for Depression and Dementia Prevention and Treatment emphasized the provision of postdiagnosis dementia care to PwD and mild cognitive impairment [10]. As China represents the largest LMIC country worldwide, the progress of dementia prevention and control policies in this country can serve as a valuable reference for other developing countries. Despite comprehensive searches in Chinese and English databases, including CNKI, WANFANG DATA, PubMed and Web of Science, using keywords such as “China”, “dementia” and “policy”, the available literature on China’s dementia prevention and control policies remains scarce. Specifically, there is a conspicuous lack of systematic reviews of these policies.

The WHO has issued the Global Action Plan on the Public Health Response to Dementia 2017–2025, which covers seven action areas: dementia as a public health priority; dementia awareness and friendliness; dementia risk reduction; dementia diagnosis, treatment, care, and support; support for dementia carers; information systems for dementia; and dementia research and innovation. This plan offers a comprehensive framework for addressing the global challenge of dementia. This study drew on these seven action areas to systematically analyse the progress of national policies for dementia prevention and control in mainland China. We intend to answer the following three questions:

(i) What are the characteristics of China’s dementia prevention and control policies?

(ii) To what extent do the current policy documents address the concerns of the WHO global action plan on dementia?

(iii) How are related departments coordinated according to the policy documents?

The findings of this research can greatly enrich our understanding of policy development pertaining to dementia prevention and control in China and particularly inform further policy design for other LMIC countries.

## Methods

### Data sources and search strategy

The main sources of information for this review included the official websites of government departments: (1) the State Council, (2) the National Health Commission (NHC), (3) the National Ministry of Civil Affairs (MCA), (4) the National Development and Reform Commission (NDRC), (5) the National Healthcare Security Administration (NHSA) and (6) the National Administration of Traditional Chinese Medicine (NATCM). All search sites and search strategies can be found in Supplementary File 1.

The policy documents pertaining to dementia prevention and control included in this study were released to the public by 15 April 2023. The search terms included *Chi Dai* (dementia), *A Er Ci Hai Mo* (Alzheimer’s), *Ren Zhi Zhang Ai* (cognitive impairment) and *Shi Zhi* (dementia), which are keywords used to identify dementia prevention and control policy texts in Chinese.

### Eligibility criteria

Eligible policy documents met the following inclusion criteria: (i) type of document: decision, order, announcement, note, opinion, notice and (ii) document content: must explicitly state that it is relevant to dementia prevention and control. We excluded the document types of approval, response, recommendation, and news.

### Data extraction

The data extraction process was divided into three stages. During stage one, one researcher (J.Z.) searched all information sources. In the second stage, two reviewers (J.Z. and S.Y.) screened all policy titles and removed duplicates. The whole text of all potentially acceptable policies was reviewed independently by two reviewers (J.Z. and S.Y.) in the third stage. Any disputes were discussed with the third reviewer (X.W.) to reach agreement. Name, source, date of publication, type, departments participating in policy creation and perspectives on dementia prevention and control were extracted using a prepared data extraction template in Microsoft Excel for Office 365 (version 1908).

### Theoretical framework

In 2017, the WHO launched the Global Action Plan for public health to address dementia [5]. The plan outlined seven key areas of action that countries should follow to tackle dementia. Each of these areas could be further classified into specific domains on the basis of the detailed description of the work content in each of them, resulting in a comprehensive analytical framework for policies related to dementia prevention and control (Table 1). On the basis of this framework, an analysis of the inclusion of dementia prevention and control policies could be conducted to explore the coverage and distribution of existing policies. This analysis could help identify the direction of action and the specific measures required to implement policies related to dementia prevention and control within each of the identified domains.

**Table 1:** Theoretical framework for national policies for dementia prevention and control

Theme	Subtheme
Dementia as a public health priority	Develop a policy, strategy or action plan
	Establish relevant departments
	Give human resource and financial support
	Other
Dementia awareness and friendliness	Raising awareness of dementia
	Reduce discrimination against people with dementia
	Create a community-friendly environment
Dementia risk reduction	Provide appropriate interventions
	Promote dementia risk factors
Dementia diagnosis, treatment, care and support	Screening
	Diagnosis
	Treatment
	Rehabilitation care
	End-of-life care
	Mobile device support
Support for dementia carers	Provide knowledge and skills training
	Provide respite care for PwD
	Develop policies or legislation to protect caregiver rights
Information systems for dementia	Information sharing among medical and healthcare systems
	Data related to epidemiology or caregiving
	Other
Dementia research and innovation	Research dementia
	Increase investment in dementia research
	Promote technological innovation

### Data analysis

#### Policy diffusion analysis

Policy diffusion refers to the process by which a new policy is transmitted to different regions or different levels of government through a specific mode of dissemination and is adopted and promoted. Research on policy diffusion often focusses on the motivation, constraints, patterns and characteristics of this process. This study analysed the process and characteristics of policy diffusion for dementia prevention and control in terms of two dimensions: intensity and speed [11]. The dimensions and measures of policy diffusion analysis are presented in Table 2.

**Table 2:** Dimensions and measures of policy diffusion analysis

Dimension	Basic connotation	Measurement indicator	Measurement method
Policy diffusion intensity	Within the policy sample set, the frequency of diffusion at the path level	Absolute intensity	The total frequency of individual policies being referenced, expressed as Ni
		Relative intensity	Ni/Ci, where Ci denotes the sum of the total frequency of policies being referenced in the policy reference network
Policy diffusion speed	Speed of policy diffusion to target	Intensity speed	Ni/Yi, where Yi indicates the number of years the policy was enacted

#### Policy reference network analysis method

The policy reference network analysis method was used to investigate the diffusion patterns of policies by analysing their interreferential relationships. The terminology used in policies to reflect such relationships included “based on”, “according to”, “implemented”, “forwarded”, “in accordance with” and “implemented” [12]. After using these keywords to identify the reference relationships among policies, a policy reference network could be constructed.

#### Thematic framework analysis

On the basis of Table 1, two researchers coded and analysed all the included policy contents to explore the coverage and distribution of existing policies and to identify the direction of action and specific measures stipulated by dementia prevention and control policies in each area. The types of policies, the areas covered and the existing problems were analysed.

### Synthesis of results

The data synthesis process was conducted through four steps. First, basic data elements from all eligible policy documents were systematically indexed and summarized using Microsoft Excel 2016. Second, interconnections between policy documents were identified through analysis of legislative rationales explicitly stated in the background section of each policy. Third, the cooperative relationship between the departments was confirmed according to the issuing department of the policy document. When a policy document was jointly issued by multiple departments, it is considered that there was a cooperative relationship between these departments. Fourth, the dementia-related content in the policy documents was encoded and mapped into the above analysis framework. If a policy document involved more than one of these seven areas, each area was counted separately.

**Fig. 1:**
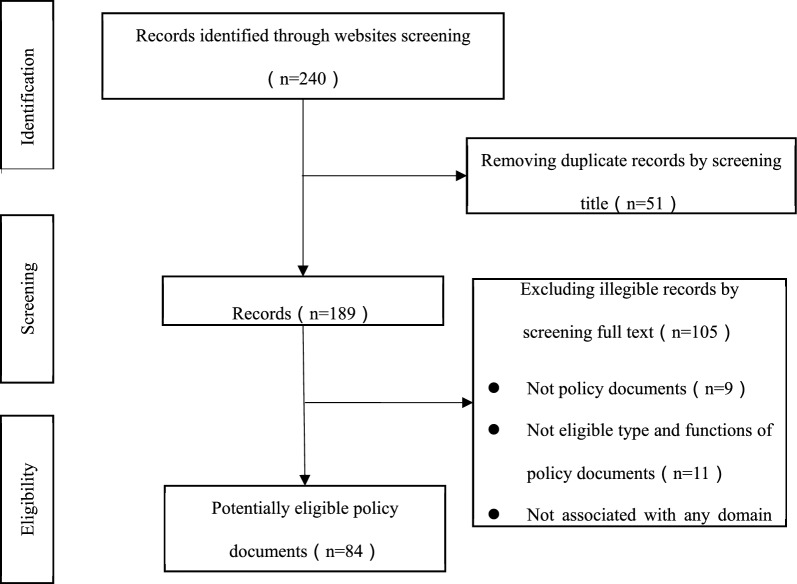
Flow chart of dementia prevention and control policy retrieval and inclusion

A total of 240 policy documents were initially retrieved in this study, and 84 policy documents were ultimately included in the analysis (Fig. 1). All the included policies can be found in Supplementary File 2.

## Results

### Characteristics of the included policies

**Fig. 2:**
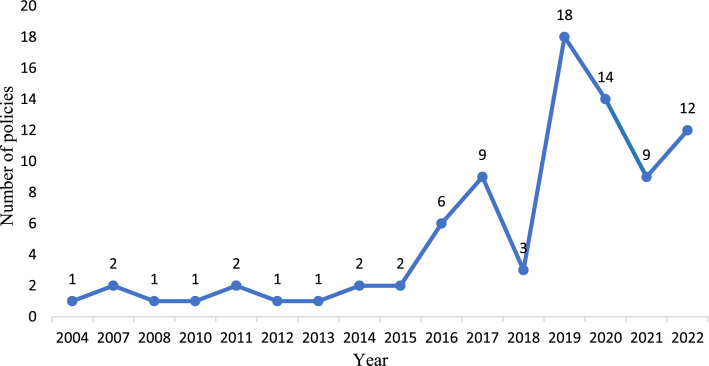
Annual distribution of dementia prevention and control policies in China

From 2004 to 2015, the issuance of dementia prevention and control-related policies in China remained relatively stable. However, a significant upward trend emerged in 2016, culminating in two distinct peaks in 2017 and 2019 (Fig. 2). This notable increase appears to be closely associated with the introduction of two pivotal national policy frameworks: the Outline of “Healthy China 2030” and Healthy China Action 2019–2030. These landmark policies established national guidelines, serving as strategic blueprints for dementia prevention and control initiatives.

In detail, the Outline of “Healthy China 2030”, issued in 2016 by the State Council, represented the first time that dementia prevention and control had been elevated to a higher strategic level. It strengthened the consensus of the entire society regarding the development of a healthy China. Various departments and organizations introduced additional policies to ensure the successful implementation of the Healthy China strategy. To enhance and solidify the top-level design, the Healthy China Action Promotion Committee established the Healthy China Action 2019–2030 in July 2019 [13]. This action plan highlighted the importance of preventing, detecting and treating dementia. Moreover, it also established the goals of reducing disability rates among individuals aged 65–74 years and decreasing the incidence of dementia among individuals aged 65 years or older by 2022 and 2030, respectively.

Among the 50 departments involved in policy-making, the NHC exhibited the highest frequency of participation, followed by the MCA, the Ministry of Finance (MOF) and the State Council. The top ten departments in terms of publication frequency are shown in Supplementary File 3.

### Analysis of the interrelationships between dementia prevention and control policies

#### Collaboration among different issuing departments

**Fig. 3:**
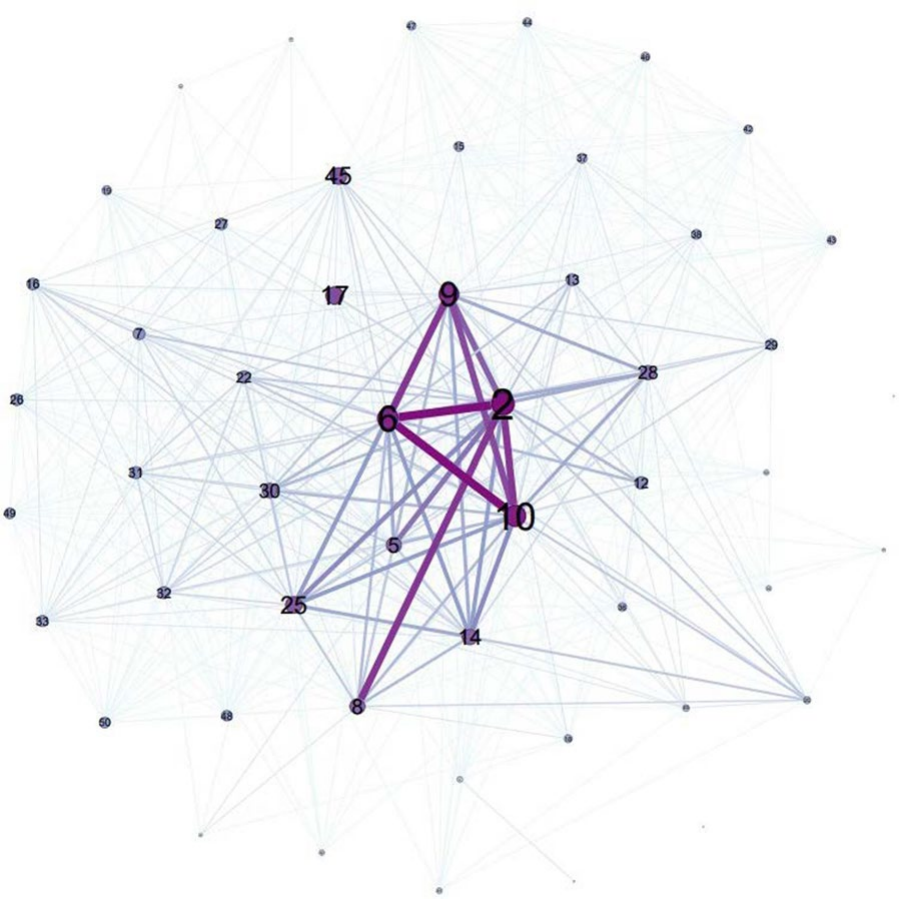
Network of departments involved in the co-development of dementia prevention and control policies. Notes: 2—NHC; 5—Office of the National Committee on Ageing Work; 6—MCA; 8—NATCM; 9—NDRC; 10—MOF; 14—Ministry of Human Resources and Social Security (MOHRSS); 17—Ministry of Science and Technology of the People’s Republic of China (MOST); 25—Ministry of Education (MOE); 28—Ministry of Housing and Urban–Rural Development (MOHRUD); 30—China Disabled Persons’ Federation; 45—Ministry of Culture and Tourism

The collaborative relationships among these departments are shown in Fig. 3. A total of 31 policy documents were jointly developed by two or more departments, with the Guidance on Strengthening Mental Health Services issued in 2017, exhibiting the highest number of collaborating departments (*N* = 22). The most common combinations of issuing departments were the NHC and the MCA (*N* = 14) and the MCA and the MOF (*N* = 14), followed by the NATCM and the NHC (*N* = 12) and the MOF and the NHC (*N* = 12).

#### Reference relationships among policies: policy diffusion analysis

Of the 84 policies pertaining to dementia prevention and control included in the study, a total of 12 policies were referenced by other policies. Table 3 presents the diffusion intensity and diffusion speed of these policies. Amongst them, the three policies that exhibited the highest diffusion intensity were the Outline of “Healthy China 2030”, the Healthy China Action 2019–2030 and the Opinions on Promoting the Development of Elderly Care Services [14]. These policies reached absolute intensities of diffusion (relative intensities) of 9 (0.281), 8 (0.250) and 3 (0.094), respectively. In terms of diffusion rate, the only difference was that the Opinions on Strengthening the Work of the Elderly in the New Era, which was promulgated by the State Council in 2021, was ranked third. [15]

**Table 3:** List of diffusion intensity and diffusion rate of dementia prevention and control policies in China

	Document name	Department	Time	Ni	Ni/Ci	Ni/Yi
1	Outline of “Healthy China 2030”	State Council	2016	9	0.281	1.286
2	Healthy China Action 2019–2030	HCAPC	2019	8	0.250	2.000
3	Opinions on Promoting the Development of Elderly Care Services	State Council	2019	3	0.094	0.750
4	Notice on Printing and Distributing the 13th Five-Year Plan for Healthy Ageing	NHC	2017	2	0.063	0.333
5	Opinions on Strengthening the Work of the Elderly in the New Era	State Council	2021	2	0.063	1.000
6	Opinions on Further Expanding the Supply of Elderly Care Services and Promoting the Consumption of Elderly Care Services	MCA	2019	2	0.063	0.500
7	Opinions of the State Council on Implementing Healthy China Action	State Council	2019	1	0.031	0.250
8	Guidance on Strengthening Mental Health Services	NHC	2017	1	0.031	0.167
9	Notice on Printing and Distributing the 13th Five-Year Plan for Health and Health	State Council	2017	1	0.031	0.167
10	Notice on the Construction of Elderly-Friendly Medical Departments	NHC	2020	1	0.031	0.333
11	Opinions on Further Promoting the Development of a Combination of Medical Care and Nursing Care	NHC	2019	1	0.031	0.250
12	Notice on the Issuance of the 14th Five-Year Plan for the Development of the National Elderly and the Elderly Service System	State Council	2022	1	0.031	1.000

### The focus of policy contents for dementia prevention and control

**Fig. 4:**
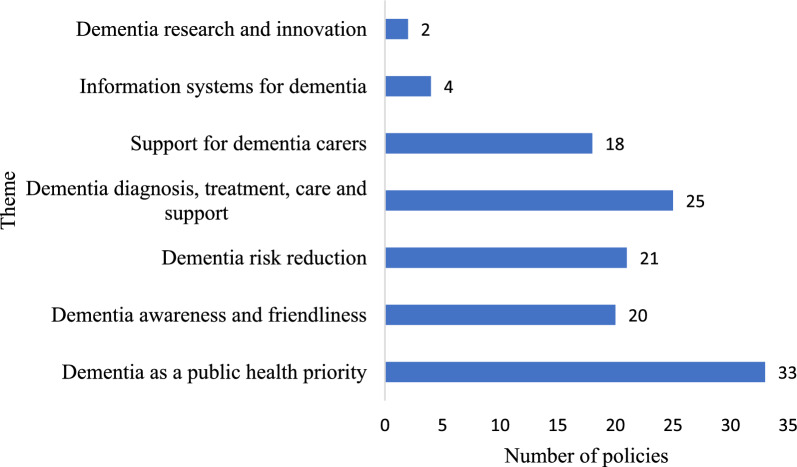
Distribution of directions covered by dementia prevention and control policies

Overall, the area associated with the highest number of implemented policies was “dementia as a public health priority” (*N* = 33), and significantly fewer policy documents were related to “information systems for dementia” (*N* = 4) and “dementia research and innovation” (*N* = 2) than to other areas (Fig. 4). The topics covered in each policy document and related content are shown in Supplementary File 4.

#### Dementia as a public health priority

- Develop a policy, strategy or action plan

A total of 19 policy documents mentioned the topic of dementia, but the degree of emphasis on dementia prevention and control varied greatly among the documents; 5 policies clearly prioritized dementia prevention and control, such as a policy that called for the development of a pilot work plan for the comprehensive prevention and treatment of dementia in the community (Notice on the Issuance of the Work Plan for Promoting the Implementation of the Health China Action 2020). A total of 14 policies outlined steps that should be taken to prioritize the prevention and control of dementia. These policies covered various aspects, such as prevention and treatment, the provision of dementia-related medical services and the establishment of care systems. Notably, the Notice on the Comprehensive Strengthening of Health Services for the Elderly [16] suggested actively conducting early screenings and providing health guidance with regard to neurodegenerative diseases such as Alzheimer’s and Parkinson’s as well as making efforts to enhance public knowledge of the prevention and treatment of dementia among elderly individuals.

(b) Establish relevant departments

In 2019, the MCA issued a policy document called the Opinions on Further Expanding the Supply of Elderly Care Services and Promoting the Consumption of Elderly Care Services. This document highlighted the importance of developing elderly care departments that meet the basic needs of elderly people and individuals with mental disorders and claimed that the government should continuously improve the nursing capabilities of these departments.

(c) Give human and financial support

A total of 11 policy documents mentioned the “give human and financial support”, with a consistent emphasis on financial assistance for the care of PwD. For instance, one policy focussed on including the provision of training to family members of people with physical or intellectual disabilities and elderly individuals in the government’s catalogue of elderly care services. Another policy prioritized the delivery of free or subsidized care services to economically disadvantaged elderly individuals with disabilities. These measures collectively underscore the government’s commitment to reducing the financial burden associated with dementia care.

A total of 11 policy documents explicitly addressed the provision of “human and financial support”, with a consistent emphasis on financial assistance for the care of PwD. For example, one policy highlighted the integration of training programs for family members of individuals with physical or intellectual disabilities, as well as elderly individuals, into the government’s official catalogue of elderly care services. Another policy prioritized the delivery of free or subsidized care services to economically disadvantaged elderly individuals with disabilities. These measures collectively underscore the government’s commitment to alleviating the financial burden associated with dementia care.

(d) Other

In addition to the elements of this area covered by the WHO action plan, we identified three other themes in this area: eight policies focussed on the issue of the acceptance of older PwD in care structures, one policy promoted the experience of caring for older PwD and one policy established a system of regular visits for older PwD.

#### Dementia awareness and friendliness

- Raising awareness of dementia

A total of 13 policy documents mentioned this content, all of which shared the goal of increasing public awareness of dementia through popular science education. Policy strategies supported in these policies included Senior Health Promotion Week, the Month of Respect for Elderly Individuals, the Chung Yeung Festival and World Alzheimer’s Disease Day. More detailed and comprehensive methods of popularizing such scientific information were provided, such as local operas, folk songs and fast-paced songs as well as using WeChat, Weibo and mobile media to promote science education.

(b) Reduce discrimination against PwD

One policy document, the Notice on the Issuance of Core Information on Alzheimer’s Disease Prevention and Intervention [17], which was introduced in 2019, stated its intention to “strengthen social awareness, reduce discrimination againstPwD, care for PwD and their families, and build a friendly social environment”.

(c) Create a community-friendly environment

A total of nine policy documents highlighted the need to create a community-friendly environment, focussing on two points. First, they greatly emphasized the need to establish a friendly atmosphere and a socially supportive environment for caring for elderly people with Alzheimer’s disease and their families. Second, they focussed on encouraging the government, the community and volunteers to assist in the lives of elderly PwD, such as by offering age-appropriate modifications or encouraging family doctors to provide home treatment, follow-up management, rehabilitation, nursing care, hospice care and health guidance to the families of elderly PwD.

#### Dementia risk reduction

- Provide appropriate interventions

A total of 16 policy documents focussed on the provision of appropriate interventions. For example, the Outline of “Healthy China 2030” mentioned the need to “promote the development of mental health and care services for the elderly and strengthen effective interventions for dementia”, and the Notice on the Issuance of the 13th Five-Year Healthy Ageing Plan Key Tasks mentioned that a prevention and intervention program for the mental health of elderly individuals should be developed, thereby providing daily care and psychological support services for elderly individuals with dementia.

(b) Promote the reduction of dementia risk factors

A total of seven policy documents addressed risk factors pertaining to dementia, only one of which specifically focussed on Alzheimer’s disease. The rest of these policies were notices regarding tobacco control education or chronic disease awareness campaigns, which listed dementia as a consequence of smoking or chronic disease.

#### Dementia diagnosis, treatment, care and support

- Screening, diagnosis, treatment

A total of nine policy documents referred to screening for dementia, six to the diagnosis of dementia and nine to the treatment of dementia. The corresponding themes were presented in the same manner in these policies; all of them highlighted the necessity and importance of carrying out this work, such as actively ensuring early screening for dementia, emphasizing a clear diagnosis of dementia, strengthening the treatment of dementia and promoting appropriate prevention and treatment techniques.

(b) Rehabilitation care

A total of eight policy documents referred to rehabilitation care for dementia; these documents clearly explicated the provision of necessary medical and care services for elderly PwD. No further specific information was provided. The care service model for PwD was also identified as a key content of these policies, including a requirement for community day care centres and township elderly homes to provide long-term care services for elderly PwD. The active development of an approach based on the principle of “internet + nursing services” was encouraged to provide convenient and professional medical nursing services for PwD. Moreover, the policy also emphasized the requirements for integrated medical and nursing departments, particularly with regard to three aspects: living care services, basic care services and rehabilitation services.

(c) End-of-life care

One policy document that mentioned end-of-life care for dementia was the Notice on Accelerating the Construction of Health and Elderly Services, which listed end-of-life care as one of the main tasks of the health service system.

(d) Mobile device support

Mobile devices used to support PwD were clearly emphasized in five policy documents. These devices fell into three main categories: cognitive impairment assessment and training aids, anti-wandering bracelets used to locate and prevent wandering and smart monitoring devices for elderly individuals, such as smart wheelchairs and monitoring beds.

#### Support for dementia carers

- Providing knowledge and skills training

A total of 14 documents mentioned the provision of knowledge and skills training to caregivers, and this content covered three areas. First, these documents emphasized the provision of care training for informal caregivers, such as “including training for family members of disabled elderly persons in the catalogue of government purchase of elderly care services”. Second, they emphasized the provision of knowledge and skills training for medical personnel and professional caregivers, such as “regular training will be provided to staff of medical and health departments at all levels in pilot areas”. Third, the diagnosis, treatment and care of dementia were included in the assessment syllabus as assessment indicators for medical personnel and were mentioned in policy documents.

(b) Providing respite care for PwD

A total of four policy documents referred to the provision of respite care services to PwD, and the contents pertaining to this theme converged. Respite services for family carers of elderly PwD were developed to reduce pressure on family carers. For example, these documents indicated that volunteers should be encouraged to provide temporary assistance to family members caring for elderly individuals with disabilities at home and to provide care services to economically disadvantaged elderly people with intellectual disabilities.

#### Information systems for dementia

- Information sharing among medical and healthcare systems

One policy explicitly mentioned the sharing of information between medical and healthcare systems. The Notice on Exploring Special Services for Depression and Dementia Prevention and Treatment, which was issued in 2020 mentioned the establishment of an information-sharing service platform that includes modules for promoting scientific knowledge, accessing service resources, managing patient treatment and exploring mechanisms for information sharing and communication among pilot regions.

(b) Data related to epidemiology or caregiving

One policy explicitly mentioned data related to epidemiology or care, and the Notice on the Issuance of the Mental Health Work Indicators Survey Assessment Program, which was issued in 2010, identified the early detection rate and early intervention rate for dementia as indicators of mental health work; this policy also provided the relevant sample and sampling method.

#### Dementia research and innovation

Two policies explicitly mentioned conducting dementia-related research, although their research directions were not entirely consistent. One policy mentioned that three-tier general hospitals should strengthen clinical research on the integration of traditional Chinese medicine and Western medicine with a focus on Alzheimer’s disease and the prevention and treatment of high-altitude diseases. The 13th Five-Year Plan for Health and Health Science and Technology Innovation focussed on the role of exercise in the cognitive impairment resulting from neurodegenerative diseases from a basic medical perspective.

## Discussion

This scoping review shows that the number of national policies for dementia prevention and control in mainland China consistently increased during the period of 2015–2022. The NHC issued the largest number of policies and the State Council issued the most important policies. Although 50 departments were involved in the development of policies for dementia prevention and control, most government departments were involved in such development very few times and did not have relatively stable collaborative relationships. On the basis of the seven areas proposed in the WHO dementia global action plan, the included policies particularly emphasized “dementia as a public health priority” and “diagnosis, treatment, care and support for dementia”, but were less focussed on “dementia research and innovation” and “information systems for dementia”.

First, regarding diffusion intensity and diffusion speed, the Outline of “Healthy China 2030” (Ni = 9, Ni/Ci = 0.281, Ni/Yi = 1.286) and Healthy China Action 2019–2030 (Ni = 8, Ni/Ci = 0.250, Ni/Yi = 2.000) demonstrated significantly higher metrics compared with other policies. This can likely be attributed to their status as critical national-level strategic plans, which established top-level designs and provided guiding principles for subsequent dementia prevention and control policies. Consequently, these two policies were frequently referenced by various departments when formulating related policies. This pattern suggests that policies with high diffusion intensity serve as pivotal drivers and exemplars within the policy system, exerting a leading influence on policy development. Guided by the Outline of “Healthy China 2030”, numerous policies revolve around enhancing the mental health of the elderly and strengthening dementia prevention and control interventions. This reflects that policy-making is closely aligned with the overall national health development goals, aiming to promote the integration of dementia prevention and control work into the national health system construction from a strategic perspective.

Among all departments involved in the development of dementia prevention and control policies, only a limited number of agencies, including the NHC, the MCA, the MOF and the NATCM, demonstrated sustained engagement in policy formulation and maintained collaborative relationships with other departments. The NHC emerged as the most active participant in policy-making, assuming a central and leading role, particularly in areas such as medical services and disease prevention and control. The State Council, on the contrary, plays a leading role in the top-level design and important decision-making of policies. The policies issued by the State Council are authoritative and instructive, setting the tone for the entire prevention and control work.

Due to the high social–economic burdens caused by dementia, dementia prevention and control requires joint efforts on the part of multiple departments in the whole society, such as health, livelihood and finance. Therefore, the development and implementation of dementia prevention and control policies require the participation and collaboration of multiple departments. The global action plan also highlighted the importance of multidepartment collaboration.

Second, regarding the focus of the policy content, the areas associated with the most policy content were “making dementia a public health priority” (*N* = 33) and “diagnosis, treatment, care and support for dementia” (*N* = 25). This situation may be related to the Outline of “Healthy China 2030”, which highlighted the needs to “promote the development of mental health and care services for the elderly and strengthen effective interventions for dementia”, thus emphasizing the prevention and treatment of the disease and focusing on PwD. In addition, the WHO’s Dementia: A Public Health Priority issued in 2012, also focussed on the diagnosis and care of dementia and proposed a “seven-stage model for dementia service planning”, which divided the whole process of dementia services into seven stages according to the period preceding diagnosis as well as the early, middle and late stages of a dementia diagnosis. However, the topics regarding information system construction and scientific research and innovation emerged less. These findings remind us of the future focus of national policies pertaining to dementia prevention and control.

In comparison, some developed countries implemented national-level dementia policies or programs long before the WHO issued the global action plan, such as Japan’s Emergency Project for Improvement of Medical Care and Quality of Life for People with Dementia, which was introduced in 2008 and has since been implemented, and Australia’s The Dementia Initiative: Making Dementia a National Health Priority, which was implemented from 2005 to 2011. Furthermore, the French Alzheimer’s Disease Plan was implemented in France from 2008 to 2012. These plans focussed on ensuring the early diagnosis of dementia, increasing awareness of dementia, enhancing caregiver support and improving service quality. [18]

In comparison with dementia policies in Japan, Australia and France, China shares similar objectives, such as improving prevention and treatment and reducing the burden on PwD and their families. However, taking into account its huge elderly population base and the trend of fast ageing, China has clearly set specific quantitative targets in the Healthy China Action 2019–2030 to reduce the incidence of disability and the prevalence of dementia in specific age groups. In terms of strategies, Japan emphasizes scientific research and early diagnosis [19], Australia focusses on training elderly care workers and supporting patients and families [20] and France strengthens caregiver support and intersectoral coordination [21]. In contrast, China prioritizes building grassroots capacities, such as enhancing screening and training primary medical staff, addressing its regional disparities and urban–rural divides. This localized approach distinguishes China’s strategy from those of the other countries.

Third, although a number of policies in the area of “dementia as a public health priority”, “dementia diagnosis, treatment, care and support” and “dementia risk reduction” were emphasized, the content of these policies was mostly programmatic and lacked support from specific guidelines. This lack of specific content hindered policy implementation in practice, which also led to great discrepancies among different regions. For example, the Chinese government launched a policy pertaining to the integration of medical care and nursing care with the goal of creating a multilevel elderly care service system. However, this process is currently impeded by a significant gap in the demand for specialized dementia care, inadequate caregiving capacity and financial, work and health pressures on family caregivers. [22–27]

Lastly, we must also be aware of the gap between the policies implemented and their practical effects. There have been policy documents clearly put forward to raise the public awareness of the prevention and treatment of dementia to 80%. However, one study on the level of dementia awareness exhibited by WeChat users showed that almost two thirds of the participants (63.14%) were able to accurately identify risk factors. Individuals who were older than 60 years old, had low levels of education and lived in rural areas exhibited significantly lower levels of dementia knowledge [28], thus leaving a great deal of room for improvement in light of the goal of an 80% rate of public awareness. Further dissemination and education of dementia-related knowledge should be implemented to improve the current state of awareness, and elderly individuals in particular should be the target audience. In addition, several studies have found that primary healthcare professionals in China are not prepared to fully implement the dementia prevention and control policies that have been stipulated due to an inadequate knowledge base, heavy workloads and a lack of incentives [29]. Therefore, in addition to the development of new policies, ensuring the implementation of the released policies in practice required more effort on the part of the government.

One important strength of this study is its systematic summarization of policies related to dementia prevention and control in China, which enables us to identify the process by which such policies are formed and diffused as well as their characteristics, thus helping clarify the construction of policies related to dementia prevention and control in China. We also need to pay attention to the limitations of this study. Although we tried to search the national policies as much as we can, there was still a possibility of some policies being left out. We ensure that all the key departments have been searched to reduce bias. Second, due to the insufficient prior research on this topic, we focus on the policy analysis at the national level in this study. Future studies could further analyse the dementia-related policies at the provincial level on the basis of our findings.

## Policy implications

This study proposes the following three policy implications. First, policy coverage should be expanded with a focus on rural, remote and economically disadvantaged regions. This could be achieved through initiatives such as implementing online educational programs to raise public awareness and equipping primary healthcare facilities with essential screening tools and medications. Furthermore, policy scope should be extended beyond medical and nursing domains to encompass comprehensive support systems, including employment assistance and legal protection mechanisms. Second, it is crucial to delineate departmental responsibilities and establish robust evaluation frameworks. The NHC, MCA and MOF should coordinate their efforts while developing systematic monitoring and evaluation systems to track policy implementation progress and achievement of dementia prevention objectives. Third, interdepartmental collaboration should be strengthened through institutional mechanisms. This could involve establishing task forces for critical issues, developing integrated information-sharing platforms and incorporating cross-departmental cooperation effectiveness into performance evaluation metrics to incentivize active interagency collaboration.

## Conclusions

As the population ages, the pressure on the healthcare system caused by people with dementia is increasing significantly. To achieve the goal of “healthy China”, China has continued to implement policies to strengthen the prevention and control of dementia and has prioritized this issue as a public health matter. However, the policy gaps still remain at the domains of priority arrangement, multisectoral cooperation and policy implementation in practice.

## Supplementary Information

Supplementary File 1. The search strategy of government websites. File 2. The essential data items of all eligible policies. File 3. The topics covered in each policy document and related content. File 4. The policy source of departments affiliated to the State Council of China. File 5. Policy-making bodies

## Data Availability

No datasets were generated or analysed during the current study.
