# Supplementary material for: A scoping review of national policies for dementia prevention and control in mainland China
Source: Health Res Policy Syst. 2025 Apr 3;23:42. doi: 10.1186/s12961-025-01314-y (PMC11966917; doi:10.1186/s12961-025-01314-y)
Supplement: Supplementary file 1 — The search strategy of government websites. File 2. The essential data items of all eligible policies. File 3. The topics covered in each policy document and related content. File 4. The policy source of departments affiliated to the State Council of China. File 5. Policy-making bodies [file 12961_2025_1314_MOESM1_ESM.pdf]

Supplementary File 1

The search strategy of government websites

name_id	name_eng	url_searchsource_eng	search_id	search_term
1	State Council of PRC	Search	1	1. search box: Chi Dai<br>2. Search Scope: Full<br>3. Search method : full text<br>4. Sorting method : by relevance
1	State Council of PRC	Search	2	1. search box: A Er Cu Hai Mo<br>2. Search Scope: Full<br>3. Search method : full text<br>4. Sorting method : by relevance
1	State Council of PRC	Search	3	1. search box: Ren Zhi Zhang Ai<br>2. Search Scope: Full<br>3. Search method : full text<br>4. Sorting method : by relevance
1	State Council of PRC	Search	4	1. search box: Shi Zhi<br>2. Search Scope: Full<br>3. Search method : full text<br>4. Sorting method : by relevance
2	National Health Commission	Advanced search	5	1. Keywords: Chi Dai<br>2. Time scale: All time<br>3. Sorting method : by relevance<br>4. Search Location: Anywhere
2	National Health Commission	Advanced search	6	1. Keywords: A Er Cu Hai Mo<br>2. Time scale: All time<br>3. Sorting method : by relevance<br>4. Search Location: Anywhere
2	National Health Commission	Advanced search	7	1. Keywords: Ren Zhi Zhang Ai<br>2. Time scale: All time<br>3. Sorting method : by relevance<br>4. Search Location: Anywhere
2	National Health Commission	Advanced search	8	1. Keywords: Shi Zhi<br>2. Time scale: All time<br>3. Sorting method : by relevance<br>4. Search Location: Anywhere
3	Ministry of Civil Affairs	Advanced search	9	1. Keywords: Chi Dai<br>2. Time scale: All time<br>3. Sorting method : by relevance<br>4. Search Location: Anywhere

3	Ministry of Civil Affairs	Advanced search	10	1. Keywords: A Er Cu Hai Mo<br>2. Time scale: All time<br>3. Sorting method : by relevance<br>4. Search Location: Anywhere
3	Ministry of Civil Affairs	Advanced search	11	1. Keywords: Ren Zhi Zhang Ai<br>2. Time scale: All time<br>3. Sorting method : by relevance<br>4. Search Location: Anywhere
3	Ministry of Civil Affairs	Advanced search	12	1. Keywords: Shi Zhi<br>2. Time scale: All time<br>3. Sorting method : by relevance<br>4. Search Location: Anywhere
4	National Development and Reform Commission	Advanced search	13	1. Keywords: Chi Dai<br>2. Time scale: All time<br>3. Sorting method : by relevance<br>4. Search Location: Anywhere
4	National Development and Reform Commission	Advanced search	14	1. Keywords: A Er Cu Hai Mo<br>2. Time scale: All time<br>3. Sorting method : by relevance<br>4. Search Location: Anywhere
4	National Development and Reform Commission	Advanced search	15	1. Keywords: Ren Zhi Zhang Ai<br>2. Time scale: All time<br>3. Sorting method : by relevance<br>4. Search Location: Anywhere
4	National Development and Reform Commission	Advanced search	16	1. Keywords: Shi Zhi<br>2. Time scale: All time<br>3. Sorting method : by relevance<br>4. Search Location: Anywhere
5	National Healthcare Security Administration	Advanced search	17	1. Keywords: Chi Dai<br>2. Search category: All<br>3. Document format: Any format<br>4. Search scope: All<br>5. Sorting method : by relevance
5	National Healthcare Security Administration	Advanced search	18	1. Keywords: A Er Cu Hai Mo<br>2. Search category: All<br>3. Document format: Any format<br>4. Search scope: All<br>5. Sorting method : by relevance

5	National Healthcare Security Administration	Advanced search	19	1. Keywords: Ren Zhi Zhang Ai<br>2. Search category: All<br>3. Document format: Any format<br>4. Search scope: All<br>5. Sorting method : by relevance
5	National Healthcare Security Administration	Advanced search	20	1. Keywords: Shi Zhi<br>2. Search category: All<br>3. Document format: Any format<br>4. Search scope: All<br>5. Sorting method : by relevance
6	National Administration of Traditional Chinese Medicine	Advanced search	21	1. Search Scope: Unlimited<br>2. Time Limit: Unlimited<br>3. Results Display: according to the release date，in reverse order<br>4. Keyword: Chi Dai
6	National Administration of Traditional Chinese Medicine	Advanced search	22	1. Search Scope: Unlimited<br>2. Time Limit: Unlimited<br>3. Results Display: according to the release date，in reverse order<br>4. Keyword: A Er Cu Hai Mo
6	National Administration of Traditional Chinese Medicine	Advanced search	23	1. Search Scope: Unlimited<br>2. Time Limit: Unlimited<br>3. Results Display: according to the release date，in reverse order<br>4. Keyword: Ren Zhi Zhang Ai
6	National Administration of Traditional Chinese Medicine	Advanced search	24	1. Search Scope: Unlimited<br>2. Time Limit: Unlimited<br>3. Results Display: according to the release date，in reverse order<br>4. Keyword: Shi Zhi

Supplementary File 2

The essential data items of all eligible policies

ID	Policy name (English)	Source	Issue date	Co-developed policies	Number of departments	Seven domains of global action plan for dementia (1 - included, 0 - not included)						
						Dementia as a public health priority	Dementia awareness and friendliness	Dementia risk reduction	Dementia diagnosis, treatment, care, and support	Support for dementia carers	Information systems for dementia	Dementia research and innovation
100001	Notice on exploring special services for depression and dementia prevention and treatment	State Council	2020. 09.11	No	1	1	1	1	1	1	1	0
100002	Notice on the issuance of core information on Alzheimer's disease prevention and intervention	State Council	2019. 11.15	No	1	0	1	1	0	1	0	0
100003	Notice on the implementation of psychologicalcare project for the elderly	NHC	2019. 04.02	No	1	0	0	0	1	0	0	0
100004	Notice on the 2019 National Alzheimer's Disease Prevention and Intervention Campaign	NHC	2019. 09.19	No	1	0	1	0	0	0	0	0
100007	Notice on the issuance of the Core Messages and Knowledge Essentials for Mental Health Publicity and Education	NHC	2007. 07.02	No	1	1	1	0	0	0	0	0
100008	Notice on Forwarding the National Mental Health Work Plan (2015-2020) of the Health and Family Planning Commission and Other Departments	NHC	2015. 06.18	No	1	1	0	0	0	0	0	0
100009	Notice on the issuance of core information on the prevention of disability in old age	NHC	2019. 08.27	No	1	0	1	0	0	0	0	0
100010	Notice on the issuance of core information on tobacco control health education	NHC	2013.08.2 2	No	1	0	0	1	0	0	0	0
100011	Notice on the issuance of the mental health work indicators survey assessment program	NHC	2010. 03.08	No	1	0	1	0	0	0	0	0
100012	Notice on the issuance of the "Primary Health Care Institutions General Practitioner Transfer	NHC	2011. 01.11	No	1	0	0	0	0	1	0	0

	Training Syllabus (for Trial Implementation)											
100014	Notice on the comprehensive strengthening of health services for the elderly	NHC	2022.01.17	Yes	3	1	1	0	1	0	0	0
100016	Notice on the issuance of the 13th five-year Healthy Aging Plan key tasks division of labor	NHC	2017.11.15	No	1	1	0	1	1	0	0	0
100017	Notice on the issuance of pilot areas of psychosocial service system training program for grass-roots personnel	NHC	2020.12.15	No	1	0	0	0	0	1	0	0
100018	Notice on the issuance of the Health Angel Youth Volunteer Service Action Plan (2018-2020)	NHC	2018.10.20	Yes	2	0	0	1	0	0	0	0
100019	Notice on the issuance of Chinese Citizens' Health Literacy - Basic Knowledge and Skills (2015 Edition)	NHC	2016.01.06	No	1	0	1	0	0	0	0	0
100020	Notice on the Issuance of the General Practitioner Transfer Training Syllabus (2019 Revised Edition)	NHC	2019.04.02	No	1	0	0	0	0	1	0	0
100021	Notice on the Solicitation of Opinions on the Identification and Management Measures of Standardized Training Bases for General Practitioners (Draft for Comments)	NHC	2012.01.30	No	1	0	0	0	0	1	0	0
100022	Notice on the Issuance of Job Training Syllabus for Community Health Personnel	NHC	2007.09.28	No	1	0	0	0	0	1	0	0
100023	Opinions on Further Strengthening the Work of Chinese Medicine in General Hospitals to Promote the Synergistic Development of Chinese and Western Medicine	NHC	2021.06.30	Yes	3	0	0	0	0	0	0	1
100024	Notice on the 28th World No Tobacco Day and Healthy China Walk - 2015 Tobacco Free Life Theme Publicity and Education Activities	NHC	2015.05.22	Yes	7	0	0	1	0	0	0	0
100025	Opinions of the State Council on implementing healthy China action	NHC	2019.07.15	No	1	1	0	0	0	0	0	0
100026	Notice on the issuance of the Guideline for the	NHC	2008.	Yes	17	0	1	0	0	0	0	0

	Development of the National Mental Health Work System (2008-2015)		04.28									
100027	Opinions on strengthening the work of the elderly in the new era	NHC	2021. 11.24	Yes	2	1	0	1	0	0	0	0
100028	Notice on the issuance of the work plan for promoting the implementation of the Health China Action 2020	NHC	2020. 09.01	No	1	1	1	0	0	0	0	0
100029	Notice on printing and distributing the 13th five-year plan for healthy aging	NHC	2017. 03.17	Yes	13	1	0	1	1	0	0	0
100030	China's 12th five-year Plan for the Development of Aging	NHC	2011. 11.23			1	0	0	0	0	0	0
100031	Outline of "Healthy China 2030"	NHC	2016. 10.26	Yes	2	0	0	1	0	0	0	0
100032	On the issuance of the national psychosocial service system construction pilot work program notice	NHC	2018. 12.04	Yes	10	0	0	0	1	0	0	0
100033	Healthy China action 2019-2030	NHC	2019. 07.15	No	1	1	1	1	1	1	0	0
100034	Guidance on strengthening mental health services	NHC	2017. 01.19	Yes	22	0	0	1	1	0	0	0
100035	Notice on printing and distributing the 13th five-year plan for health and health	NHC	2017. 01.10	No	1	1	0	1	0	0	0	0
100036	Outline of the 13th five-year Plan and Vision 2010 for China's Health Science and Technology Development	NHC	2004. 06.03			0	0	0	1	0	0	0
100041	Notice on printing and distributing the national disability prevention action plan (2016-2020)	NHC	2016. 09.07	No	1	0	0	0	1	0	0	0
100042	Notice on the promotion of World Stroke Day 2022	NHC	2022. 10.21	No	1	0	0	1	0	0	0	0
100043	Notice on the issuance of the work plan for the demonstration project of the integration of health care	NHC	2022. 04.27	No	1	1	0	0	0	1	0	0

100045	Notice on the collection and promotion of typical experience in the integration of health care	NHC	2019.07.26	No	1	1	0	0	0	0	0	0
100046	Notice on the promotion of World Stroke Day 2020	NHC	2020.10.15	No	1	0	0	1	0	0	0	0
100047	Notice on the promotion of World Stroke Day 2019	NHC	2019.10.10	No	1	0	0	1	0	0	0	0
100048	Notice on the guiding opinions on promoting the development of elderly products industry	NHC	2020.01.21	Yes	5	0	0	0	1	0	1	0
100049	Notice on the issuance of the content of the national grassroots health job training and skills competition and related matters	NHC	2016.09.28	No	1	0	0	1	0	0	0	0
100050	Notice on the launch of the 2021 Chronic Disease Publicity Day Series	NHC	2021.09.18	No	1	0	0	1	0	0	0	0
100051	Notice on the Issuance of the Technical Plan for Graded Treatment of Coronary Atherosclerotic Heart Disease and Cerebrovascular Disease	NHC	2017.02.09	Yes	2	0	0	0	1	0	0	1
100052	13th Five-Year Plan for Health and Health Science and Technology Innovation	NHC	2017.06.07	Yes	6	0	0	0	0	0	0	0
100053	Notice on the Issuance of Basic and Recommended Standards for Medical Service Capacity of County Hospitals	NHC	2016.05.12	No	1	0	0	0	1	0	0	0
100055	Notice on the Creation of Model National Age-Friendly Communities	NHC	2020.12.11	Yes	2	1	1	0	0	0	0	0
100056	Notice on the convenience of the elderly in the primary health care institutions to see the relevant work	NHC	2021.01.08	No	1	0	0	1	1	0	0	0
100058	Notice on the fourth national "civilized number of respect for the elderly" creation activities	NHC	2021.07.06	Yes	2	0	1	0	0	0	0	0
100060	Notice on the issuance of guidelines for the management of combined medical and health institutions (for trial implementation)	NHC	2020.10.10	Yes	3	0	0	0	1	0	0	0
100061	Notice on the development of psychological care actions for the elderly	NHC	2022.06.20	No	1	0	1	0	0	0	0	0

100062	Notice on the construction of elderly-friendly medical institutions	NHC	2020.12.02	Yes	2	0	0	1	1	0	0	0
100064	Opinions on promoting the healthy development of elderly care services	NHC	2021.01.05	No	1	1	0	0	0	0	0	0
100065	Notice on the implementation of the project of health for the elderly in Western China	NHC	2019.04.08	No	1	0	1	0	0	0	0	0
100066	Notice on the in-depth implementation of the 2019 National "Month of Respect for the Elderly" activities	NHC	2019.09.02	No	1	0	1	0	0	0	0	0
100067	Notice on accelerating the construction of health and elderly services	NHC	2014.09.19	Yes	10	0	0	0	1	0	0	0
100068	Guidance on promoting the high-quality development of family doctor contract services	NHC	2022.03.15	Yes	6	0	1	0	0	0	0	0
100069	Guidance on further promoting the development of medical and health care integration	NHC	2022.07.21	Yes	11	1	0	0	0	1	0	0
100070	Opinions on promoting the development of elderly care services	NHC	2019.04.16	No	1	1	1	0	0	1	0	0
100071	Guiding Opinions on Accelerating the Implementation of Home Aging Adaptation Project for the Elderly	NHC	2020.07.15	Yes	9	0	0	0	1	0	0	0
100072	Notice on the implementation of the service quality improvement action of medical and nursing institutions	NHC	2020.12.14	Yes	2	0	0	0	0	0	1	0
100073	Guiding opinions on the establishment and improvement of the elderly health service system	NHC	2019.11.01	Yes	8	1	1	1	1	1	0	0
100074	Notice on further strengthening the nursing work of medical institutions	NHC	2020.09.02	No	1	0	0	0	1	0	0	0
100075	Notice on printing and distributing the national nursing development plan (2021-2025）	NHC	2022.05.07	No	1	0	0	0	1	0	0	0
100077	Opinions on further promoting the development of combination of medical care and nursing care	NHC	2019.10.25	Yes	12	1	0	0	0	0	0	0
100079	Notice on the Issuance of the Outline for the Development of Chinese Women and the Outline	NHC	2021.09.27	No	1	0	0	0	0	1	0	0

	for the Development of Chinese Children											
100080	Notice on the issuance of the 14th five-year plan for the development of the national elderly and the elderly service system	MCA	2022.02.22	No	1	1	0	1	1	0	0	0
100081	Action Plan for the Development of the Smart Health and Aged Care Industry (2017-2020)	MCA	2017.02.20	Yes	3	0	0	0	1	0	0	0
100082	Notice on the pilot project of information for the elderly services and community services for the benefit of the people (repealed)	MCA	2014.10.03	Yes	6	0	0	0	0	0	1	0
100084	Notice on the issuance of the National Disability Prevention Action Plan (2021-2025)	MCA	2022.01.27	No	1	0	0	0	1	0	0	0
100093	Notice on the issuance of the action plan to further improve the quality of products, works and services (2022-2025)	MCA	2022.11.23	Yes	18	1	0	0	0	0	0	0
100094	Announcement on the Use of the Lottery Public Welfare Fund for the Year 2021	MCA	2022.06.28	No	1	1	0	0	0	0	0	0
100095	Notice on the issuance of the "14th five-year Plan" for the development of civil affairs	MCA	2021.06.18	Yes	2	1	0	0	0	1	0	0
100097	Notice on the Issuance of the Training Curriculum for Nursing Home Directors (for Trial Implementation) and the Training Curriculum for Elderly Social Workers (for Trial Implementation)	MCA	2020.10.23	No	1	0	0	0	0	1	0	0
100098	Notice on the Implementation of the Vocational Skills Training Program for Recreation and Wellness	MCA	2020.10.23	Yes	5	0	0	0	0	1	0	0
100099	Announcement on the Use of Lottery Public Welfare Fund in 2019	MCA	2020.06.30	No	1	1	0	0	0	0	0	0
100100	Notice on the Declaration of the Fifth Batch of Home and Community-based Elderly Services Reform Pilot	MCA	2019.11.11	Yes	2	1	0	0	0	0	0	0
100101	Opinions on further expanding the supply of	MCA	2019.	No	1	1	0	0	0	0	0	0

	elderly care services and promoting the consumption of elderly care services		09.20									
100102	Opinions on the implementation of the renovation and upgrading project of the facilities for special hardship support services (homes for the elderly)	MCA	2019. 08.21	Yes	3	1	0	0	0	0	0	0
100103	Announcement on the use of lottery public welfare funds in 2018	MCA	2019. 06.28	No	1	1	0	0	0	0	0	0
100104	Announcement on the use of the lottery public welfare fund in 2017	MCA	2018. 06.29	No	1	1	0	0	0	0	0	0
100105	Announcement of the use of the lottery public welfare fund in 2016	MCA	2017. 06.30	No	1	1	0	0	0	1	0	0
100162	The 14th five-year Plan for National Health	NDRC	2022. 06.01			1	0	0	0	0	0	0
100163	Robotics industry development plan (2016-2020)	NDRC	2016. 04.27			0	0	0	0	0	1	0
100164	Opinions on the service of "six stable" "six protection" to further improve the "management and service" reform related work	NDRC	2021. 10.29	No	1	1	0	0	0	0	0	0
100165	Notice on printing and distributing the development plan of elderly education (2016-2020)	NDRC	2017. 05.11	No	1	0	1	0	0	0	0	0

Supplementary File 3

The topics covered in each policy document and related content

Theme	Subtheme	Amount	Document	Related content
Dementia as a public health priority（33）	Develop a policy, strategy, or action plan	19	Notice on the comprehensive strengthening of health services for the elderly	(III) Strengthen the Function Maintenance of the Elderly. Strengthen the early screening, intervention, and classified guidance for key chronic diseases among the elderly population. Actively conduct early screening and health guidance for neurodegenerative diseases such as Alzheimer's disease and Parkinson's disease, and improve the public's awareness rate of knowledge about the prevention and treatment of senile dementia. Encourage regions with the conditions to carry out cognitive function screening for the elderly, identify mild cognitive impairment at an early stage, and prevent and reduce the occurrence of senile dementia. Organize and carry out pilot projects on the prevention and intervention of disability (dementia) among the elderly. Encourage provinces (autonomous regions, municipalities directly under the Central Government) with the conditions to organize provincial - level pilot projects to reduce the occurrence of disability (dementia) among the elderly.
	Establish mechanisms to protect the human rights of people with dementia	0	..	..
	Establish relevant departments or departments	1	Opinions on further expanding the supply of elderly care services and promoting the consumption of elderly care services	(3) Promote the quality and efficiency of institutional elderly care services. Focus on the rigid needs of long-term care services for the elderly and the disabled and dementia, and focus on supporting the development of elderly care institutions that meet the needs of basic elderly care services and serve the elderly and the disabled and dementia. Continuously improve the care capacity of elderly care institutions for the elderly and the disabled and dementia. Where conditions permit, the special care needs of the elderly with dementia can be targeted, and the establishment of care institutions for the elderly with dementia can be supported.<br>Further deepen the reform of public elderly care institutions, adhere to the public welfare attributes of public elderly care institutions, increase the proportion of nursing beds, clarify the construction standards of nursing beds, and focus on providing care services for the extremely poor, the elderly with economic difficulties, and the elderly with special family planning. Explore diversified business models, guide social forces to set up elderly care institutions for middle- and high-income families according to market needs, and build an all-round, multi-level and three-dimensional elderly care service system to meet the needs of diversified, convenient and personalized services. By 2022, the proportion of nursing beds in elderly care institutions shall not be less than 50%.
	Give human and financial support	11	Announcement on the Use of the Lottery Public Welfare Fund for the Year	(1) the elderly welfare projects. The project funds are 1.28185 billion yuan, which is mainly used for the senior-friendly renovation of the elderly families with special difficulties; the construction and expansion of urban elderly social welfare institutions, urban community elderly care service facilities, and support service facilities for rural destitute personnel mainly serving the elderly with life difficulties and disability

			2021	and dementia, and help elderly care institutions configure fire equipment; through government purchase services, cultivate the development of home and community elderly care service organizations and institutions, and improve the coverage rate of urban and rural home and community elderly care services; carry out elderly care class skill competition.
	Other	10	Opinions on further expanding the supply of elderly care services and promoting the consumption of elderly care services	(3) Promote the quality and efficiency of institutional elderly care services. Focus on the rigid needs of long-term care services for the elderly and the disabled and dementia, and focus on supporting the development of elderly care institutions that meet the needs of basic elderly care services and serve the elderly and the disabled and dementia. Continuously improve the care capacity of elderly care institutions for the elderly and the disabled and dementia. Where conditions permit, the special care needs of the elderly with dementia can be targeted, and the establishment of care institutions for the elderly with dementia can be supported.<br>Further deepen the reform of public pension institutions, adhere to the public welfare attributes of public pension institutions, increase the proportion of nursing beds, clarify the construction standards of nursing beds, and focus on providing care services for the extremely poor, the elderly with economic difficulties, and the elderly with special family planning. Explore diversified business models, guide social forces to set up pension institutions for middle and high-income families according to market needs, and build an all-round, multi-level and three-dimensional pension service system to meet the needs of diversified, convenient and personalized services. By 2022, the proportion of nursing beds in pension institutions shall not be less than 50%.
Dementia awareness and friendliness（20）	Raising awareness of dementia	13	Notice on exploring special services for depression and dementia prevention and treatment	(1) Strengthen science popularization and education. All pilot areas should increase the intensity of community (village) level education, raise public awareness of mental health and mental health, enhance residents' awareness of Alzheimer's disease prevention and control knowledge, and reduce prejudice and discrimination. Staff of medical institutions at all levels, senior citizens' offices, elderly care institutions, and medical-nursing institutions should combine the characteristics of patients and high-risk groups to produce prevention and education materials, so that the public can obtain relevant science popularization knowledge and service resource information free of charge. Encourage the government to purchase services and commission qualified social groups to carry out science popularization.<br>Innovate the form of missionary education, such as selecting "image ambassadors", broadcasting professional authoritative and easy-to-understand public service advertisements, popular science missionary films, series of programs, and organizing experts to write popular science books. Make use of major anniversaries or festivals such as Double Ninth Festival, World Mental Health Day, and World Alzheimer's Month in our country, and use popular communication methods such as local dramas, folk songs, and allegro, as well as WeChat, Weibo, and mobile media to conduct popular science education. By 2022, the public's awareness of Alzheimer's prevention and control knowledge will increase to 80%.
	Reduce discrimination against people with	1	Notice on the issuance of core information on	Nine, create a friendly social atmosphere. Strengthen social publicity, reduce discrimination against patients, care for patients and their families, and build a friendly social environment.

	dementia		Alzheimer's disease prevention and intervention	
	Create a community-friendly environment	9	Notice on the development of psychological care actions for the elderly	From 2022 to 2025, 1,000 urban communities and 1,000 rural administrative villages will be selected nationwide to carry out caring actions. By the end of the "14th Five-Year Plan" period, in principle, at least one community or village in every county (city, district) across the country will have elderly psychological care points. See Annex 1 for the allocation of elderly psychological care points in various places. All localities carry out mental health assessments for the elderly who are permanently aged 65 and above in elderly psychological care points as required, focusing on the elderly with special families with economic difficulties, empty nests (living alone), left-behind, disabled (dementia), and family planning.
Dementia risk reduction（21）	Provide appropriate interventions	16	Outline of “Healthy China 2030”	Promote the development of mental health and care services for the elderly, and strengthen effective interventions for Alzheimer's disease. Promote the development of long-term care services for the elderly at home, comprehensively establish a subsidy system for the elderly and disabled with financial difficulties, and establish a multi-level long-term care security system.
	Promote dementia risk factors	7	Notice on the issuance of core information on Alzheimer's disease prevention and intervention	Second, reduce the risk of disease. Middle-aged people with obesity, hypertension, diabetes, stroke, depression, hearing loss, and a family history of dementia should also control their weight, correct their hearing, and maintain healthy blood pressure, cholesterol, and blood sugar levels.
Dementia diagnosis, treatment, care, and support（25）	Screening	9	Notice on the comprehensive strengthening of health services for the elderly	(3) Strengthen the functional maintenance of the elderly. Strengthen the early screening, intervention and classification guidance of key chronic diseases in the elderly, actively carry out early screening and health guidance for neurodegenerative diseases such as Alzheimer's disease and Parkinson's disease, and improve the public's awareness of the prevention and treatment of Alzheimer's disease. Encourage places where conditions permit to carry out the elderly cognitive function screening, early identification of mild cognitive impairment, prevention and reduction of Alzheimer's disease.<br>Organize and carry out the elderly disability (dementia) prevention and intervention pilot work, encourage qualified provinces (autonomous regions and municipalities) to organize provincial-level pilot work to reduce the elderly disability (dementia) occurrence.
	Diagnosis	6	Notice on the implementation of psychological care project for the elderly	3. The evaluation results show that there is suspected early Alzheimer's disease, moderate and above psychological behavior problems and mental disorders of the elderly, it is recommended to go to the mental health clinic of the general hospital for medical treatment; if necessary, it is recommended to go to the neurology department or psychiatry for further examination, in order to make a clear diagnosis and timely treatment, to achieve early detection, early diagnosis and early treatment of the disease.

	Treatment	9	Guiding opinions on the establishment and improvement of the elderly health service system	(3) Strengthen disease diagnosis and treatment. Fully implement the policy of preferential treatment of medical services for the elderly. Medical institutions generally establish green channels for the elderly to register and seek medical treatment, optimize the medical treatment process for the elderly, and provide convenient services for the elderly to see and seek medical treatment. Carry out activities to create elderly-friendly medical and health institutions, promote medical and health institutions to carry out senior-friendly transformation, and carry out elderly-friendly services. By 2022, more than 80% of comprehensive hospitals, rehabilitation hospitals, nursing homes and primary medical and health institutions will become elderly-friendly medical and health institutions. Encourage medical and health institutions to provide home beds, visits and other door-to-door medical services for the elderly with disabilities at home. (<br>The National Health Commission, the National Development and Reform Commission, the Ministry of Finance, and the State Administration of Traditional Chinese Medicine are responsible according to the division of responsibilities)
	Rehabilitation care	8	Notice on printing and distributing the 13th five-year plan for healthy aging	5. Vigorously develop combined medical and elderly care services. Establish and improve the cooperation mechanism between medical and health institutions and elderly care institutions, and encourage various forms of contracted services and agreement cooperation. Support qualified elderly care institutions to apply for the establishment of rehabilitation hospitals, nursing homes, traditional Chinese medicine hospitals, palliative care institutions or infirmaries, nursing stations, etc. in accordance with relevant regulations, focusing on providing needed medical care and life care services for the disabled and dementia elderly.
	End-of-life care	1	Notice on accelerating the construction of health and elderly services	The main tasks of the health service system include comprehensive or specialized medical and health service facilities for public health and disease diagnosis and treatment, continuous medical service facilities for chronic disease management, postoperative rehabilitation, long-term care for persons with disabilities and dementia, and hospice care, as well as health management and consultation, health examination, traditional Chinese medicine and other characteristic health care and other health management and promotion service facilities.
	Mobile Device Support	6	Guiding Opinions on Accelerating the Implementation of Home Aging Adaptation Project for the Elderly	It is used to monitor the elderly with dementia or other mental disorders to prevent the elderly from getting lost, including anti-lost bracelets and anti-lost badges.

Support for dementia carers（18）	Provide knowledge and skills training	14	Opinions on promoting the development of elderly care services	(19) Promote the integrated development of home, community and institutional elderly care. Support elderly care institutions to operate community elderly care service facilities and provide services to the elderly at home. Include the care training of disabled family members in the government's catalogue of elderly care services, organize elderly care institutions, social organizations, social work institutions, and the International Committee of the Red Cross to carry out elderly care and emergency rescue knowledge and skills training. Vigorously develop mutual aid elderly care facilities such as rural happiness homes that can be supported by the government, run by the village, and used by farmers, and serve sustainable services.<br>Explore the "property service + elderly care service" model, and support property service enterprises to carry out various forms of elderly care services such as feeding for the elderly and regular visits. Create a "three-community linkage" mechanism, with the community as the platform, social organizations for elderly care services as the carrier, and social workers as the support, vigorously support voluntary elderly care services, and actively explore mutual assistance elderly care services. Vigorously cultivate the ranks of elderly care volunteers, speed up the establishment of a volunteer service record system, and actively explore practices such as "student community volunteer service, credit counting" and "time bank" to protect the legitimate rights and interests of volunteers. (<br>Ministry of Civil Affairs, Development and Reform Commission, Ministry of Finance, Health Commission, Ministry of Housing and Urban-Rural Development, Ministry of Education, Central Committee of the Communist Youth League, and China International Committee of the Red Cross shall be responsible for the division of responsibilities)
	Provide respite care for patients	4	Guidance on further promoting the development of medical and health care integration	(13) Strengthen the disabled care service team. Improve the care ability and level of family caregivers of the elderly with disabilities by means of emergency assistance and care skills training. Strengthen the training of medical caregivers and elderly care workers who mainly care for the elderly with disabilities. Encourage volunteer service personnel to provide respite services for family members who care for the elderly with disabilities at home. (Ministry of Human Resources and Social Security, National Health Commission, Ministry of Civil Affairs, State Traditional Chinese Medicine Bureau, Emergency Department, Central Committee of the Communist Youth League, etc. are responsible according to the division of responsibilities)
	Develop policies or legislation to protect caregiver rights	0	..	..
Information systems for dementia（4）	Information sharing among medical and healthcare systems	1	Notice on exploring special services for depression and dementia prevention and treatment	(6) Build an information sharing service platform. Each pilot area should explore the establishment of an information service platform, set up modules such as popular science knowledge promotion, service resource acquisition, and patient management and treatment, and guide patients and medical staff to actively join the platform to receive services through information exchange and push. Explore information sharing and exchange mechanisms between pilot areas.

	Data related to epidemiology or caregiving	1	Notice on the issuance of the mental health work indicators survey assessment program	(III) Samples and sampling methods.<br>The survey subjects include two categories: the elderly, the elderly's family members and their caregivers. The former refers to residents aged 55 and above, and the latter refers to family members or caregivers who live with the elderly and are responsible for caring for the elderly's daily life. The elderly and their caregivers must rule out Alzheimer's or other neurological or psychiatric disorders that affect cognition.<br>Early detection and intervention rates of Alzheimer's disease<br>(1) Relevant definitions.<br>Early detection, that is, diagnosis before Alzheimer's disease has caused severe and comprehensive cognitive impairment.<br>Early intervention, that is, reasonable intervention for patients with early detection of Alzheimer's disease, mainly refers to acetylcholinesterase inhibitors.<br>According to the results of epidemiological surveys of the elderly in urban and rural Beijing by Zhang Zhenxin in our country, among people aged 55 and above, the prevalence of early old age (defined as GDS-4) is about 0.9%.<br>(2) Measurement methods.<br>It is recommended that the senile dementia prevention and treatment pilot report the early detection rate and intervention rate of senile dementia.<br>The rough estimate method is:<br>Early detection rate: Number of all early-stage Alzheimer's patients diagnosed for the pilot/(population 55 years and older in the region x 0.9%) x 100%<br>Early intervention rate: number of patients receiving acetylcholine inhibitor medication in early Alzheimer's disease diagnosed in the pilot area/number of all early Alzheimer's patients diagnosed in the pilot area × 100%<br>Questionnaire 7: Symptoms and Prevention Knowledge Questionnaire of Common Mental Disorders in Old Age
	Other	2	Notice on the pilot project of information for the elderly services and community services for the benefit of the people (repealed)	1. Improve the information service level of elderly care institutions. Upgrade and transform the existing management information system of elderly care institutions to achieve wireless positioning help for the elderly in the institution, fall monitoring, night monitoring, intelligent analysis of elderly behavior, anti-loss of dementia elderly, video intelligent linkage, access control system linkage, LBS positioning services, consumer entertainment and other services.
Dementia research and innovation（2）	Research dementia	2	13th Five-Year Plan for Health and Health Science	Basic research on exercise and health. Carry out basic research on the impact of exercise on the structure, metabolism and function of various organs and the prevention and treatment of related diseases, explore the mechanism of action of force, sound, light, electricity and magnetism

			and Technology Innovation	on the human body, carry out basic research on the prevention and treatment of depression in young and middle-aged people and the intervention effect of exercise on cognitive impairment in neurodegenerative diseases.
	Increase investment in dementia research	0	..	..
	Promote technological innovation	0	..	..

Supplementary File 4

The policy source of departments affiliated to the State Council of China

id	Government administration
1	State Council
2	National Health Commission
3	Health China Action Promotion Committee
4	Office of the Organizing Committee of the National Grassroots Health Job Training and Skills Competition
5	Office of the National Committee on Aging Work
6	Ministry of Civil Affairs
7	Central Committee of the Communist Youth League
8	National Administration of Traditional Chinese Medicine
9	National Development and Reform Commission
10	Ministry of Finance
11	Central Military Commission Logistics Department
12	Ministry of Industry and Information Technology
13	State market regulatory administration
14	Ministry of Human Resources and Social Security
15	Ministry of Commerce
16	All-China Women's Federation
17	Ministry of Science and Technology of the People's Republic of China
18	General Administration of Sport of China
19	National Medical Products Administration
20	National Healthcare Security Administration
21	National Bureau of Disease Control and Prevention
22	Ministry of Public Security
23	Central Civilization Office Secretary Bureau
24	Office of the National Council for Love and Health
25	Ministry of Education
26	General Office of the General Administration of Press, Publication, Radio and Television
27	China Association for Science and Technology
28	Ministry of Housing and Urban-Rural Development
29	China Banking and Insurance Regulatory Commission
30	China Disabled Persons' Federation
31	Publicity Department of the Communist Party of China
32	Ministry of Justice
33	All-China Federation of Trade Unions
34	Central Committee of Political and Legal Affairs
35	National Public Complaints and Proposals Administration
36	Ministry of Land and Resources of the People's Republic of China
37	People's Bank of China
38	State Taxation Administration of The People's Republic of China

39	Ministry of Natural Resources
40	Ministry of Emergency Management of the People's Republic of China
41	Cyberspace Administration of China
42	Ministry of Transport
43	Ministry of Agriculture and Rural Affairs
44	Ministry of Culture and Tourism
45	State-owned Assets Supervision and Administration Commission of the State Council
46	All-China Federation of Industry and Commerce
47	Central Office of Comprehensive Governance
48	State Administration for Industry and Commerce of the People's Republic of China
49	Chinese Academy of Sciences

Supplementary File 5

Policy making Bodies

In China, the State Council serves as the country's highest administrative organ and is constitutionally equivalent to the Central Government. As such, it directly oversees local governments in provinces, autonomous regions, municipalities, and special administrative regions. The State Council wields the powers of administrative legislation, proposal submission, and administrative leadership. Moreover, it administers diverse aspects including domestic politics, diplomacy, national defense, finance, economy, culture, and education.

In the Chinese healthcare system, "departments" denote institutions or organizational units with distinct responsibilities and functions in healthcare. Prominent examples include the NHC, the NHSA, and the NATCM. These departments cover multiple aspects, spanning from policy-making, healthcare service provision, healthcare insurance management to industry supervision. Among these, the NHC occupies a pivotal position in operational management. It is responsible for formulating national health policies, coordinating and promoting the deepening of the medical and health system reform, formulating and implementing disease prevention and control plans, etc. It acts as a crucial force in promoting the orderly operation of the healthcare system and safeguarding the health of the public.

"Ministries and commissions" are departments under the State Council, established by the central government to fulfill national management functions. Within their respective scopes of responsibility, these departments formulate policies, implement regulations, and manage related affairs to promote the development of various national undertakings. They exert a pivotal influence on diverse sectors including the economy, society, culture, and healthcare. Through their joint efforts, they form the core framework of the national administrative management system. The NHC, the MCA, and the NDRC mentioned in manuscript all belong to the ministries and commissions. The MCA is in charge of social administrative affairs and is closely related to people's livelihood security and social governance. The main responsibility of the NDRC is to formulate and implement national economic and social development strategies, medium and long term plans, and annual plans, and to conduct macroeconomic regulation and control. Due to the close connection between the MCA and the NDRC with people's livelihood security and social welfare, they are both included in the screening.
